# General practitioner trainees’ career perspectives after COVID-19: a qualitative study in China

**DOI:** 10.1186/s12875-020-01364-x

**Published:** 2021-01-11

**Authors:** Yue Yin, Xiaotian Chu, Xinxin Han, Yu Cao, Hong Di, Yun Zhang, Xuejun Zeng

**Affiliations:** Department of General Practice (General Internal Medicine), Peking Union Medical College Hospital (PUMCH), Chinese Academy of Medical Science (CAMS) and Peking Union Medical College (PUMC), Beijing, 100730 China

**Keywords:** COVID-19, General practice, General practitioner education, Career perspectives

## Abstract

**Background:**

The coronavirus disease 2019 (COVID-19) has been a worldwide public health emergency that has put great pressure on medical workers and the medical system. General Practitioners (GPs) played an important role in controlling the epidemic, and GP trainees also took an active part in this approach. This study was to explore Chinese GP trainees’ career perspectives after COVID-19.

**Methods:**

We conducted a qualitative research study which included 12 GP trainees from three teaching hospitals in China. Semi-structured telephone interviews were conducted. Grounded theory and thematic analysis were used to code the data and identify categories and factors.

**Results:**

Eleven participants chose to continue a GP career after COVID-19, and nearly half of the participants strengthened their determination to dedicate themselves to this career. Only one participant decided to change the career choice because of interest in another specialty. Four main themes influencing GP trainees’ perceptions of career development after COVID-19 emerged from the interviews: changes of GPs’ work content in COVID-19, challenges of being a GP, psychological changes of the career, how to provide better primary care. Although some negative psychological changes existed, most of participants were inspired by role models and medical colleagues. They had more in-depth understanding of GPs’ role and responsibility during COVID-19, and exhibited intensions for self-improvement in career development, especially in public health education and self-protection in preventing infectious diseases. In addition, the wide use of telemedicine provided a new work way for GP trainees. However, challenges, such as increased workloads, low income, lack of resources in primary medical institutions, and distrust of GPs are faced by trainees during the outbreak.

**Conclusions:**

Overall, no substantial changes were seen in the career choice of GP trainees after COVID-19 outbreak. However, they were inspired and had an in-depth understanding about the GP’s work and responsibility during an epidemic. Owing to the challenges faced by the GPs, measures are needed to improve the GP education and work environment in the training phase.

**Supplementary Information:**

The online version contains supplementary material available at 10.1186/s12875-020-01364-x.

## Background

In December 2019, the coronavirus disease 2019 (COVID-19) had its first outbreak in Wuhan, China [1]. Hitherto, it has escalated into a pandemic affecting more than 200 countries on six continents. The worldwide confirmed cases of COVID-19 have recorded over 76,023,488, with 1,694,128 deaths, according to World Health Organization as of 9:39 am CET, 22 December 2020 [2]. The COVID-19 global pandemic is the most extensive to afflict humanity in a century. A serious crisis for the entire world, and a daunting challenge, it poses a grave threat to human life and health [3].

During the initial outbreak of COVID-19, the Chinese government took rapid measures to control the epidemic, and millions of medical workers grappled with the epidemic at the front line across the country [3]; From January 24 to March 8, it rallied 346 national medical teams, consisting of 42,600 medical workers and more than 900 public health professionals to the immediate aid of Hubei and the city of Wuhan [3]. In addition, except for the huge efforts made in the epicenter, communities and villages made up the first line of defense in epidemic prevention and control, a major barrier to inbound cases and local transmission [3], in which GPs are at the forefront of tackling the spread of the virus in early case detection and public health education during emergencies [4, 5]. Through all these efforts, communities and villages were turned into strongholds, securing full implementation of response measures down to the lowest level, and halted the spread of the epidemic.

General practice, as a discipline, was introduced to China in the late 1980s [6]. The GP training programs introduce the integrated concepts of primary care to GP trainees, and provide them clinical rotations in hospitals and community health centers to improve competency in clinical practice. In the last two decades, the formal training program for general practice has been established to develop a large cohort of GPs in urban and rural areas [6]. Despite support from the government, there is still a huge lack of qualified GPs [7]. Consequently, GP training programs are offered to both medical graduates and specialists in secondary or tertiary hospitals to develop qualified GPs across the country.

The outbreak of COVID-19 has brought both opportunities and challenges to GPs. During the epidemic, the work content and work ways of Chinese GPs have been changed significantly, as they focused more on breaking the chains of transmission through early intervention in the community [5], except for the routine work such as chronic disease management and medication adjustment before the epidemic [4, 5, 8]. GP trainees who were in the clinical rotations also took part in the work, and had a deep impression on the unusual experience, which may influence their understanding of the career as a GP, and also their career choices. Thus, we conducted the present study aiming to explore the Chinese GP trainees’ career perspectives after COVID-19 utilizing a qualitative methodology.

## Methods

### Settings

We conducted a qualitative descriptive study in three primary care teaching hospitals from Beijing, Shanghai, and Zhejiang province, respectively. The three hospitals are university-affiliated General Practice Teaching Base, which provide three-year standard training of general practice to students who have received a five-year undergraduate education in clinical medicine, or physicians from grassroots medical and health institutions. Training includes introduction of general concepts of primary care, followed by clinical rotations in the hospital and training in the community health care center [6]. After three-year standard training, trainees will work independently in primary health care institution, providing primary acute and continuing care to individuals and families. During the epidemic, the trainees focused more on communicable disease prevention and control in their work with GPs in community or hospitals. The teaching hospitals were chosen according to diversity in terms of geographic environment and sources of GP trainees.

### Participants and recruitment

On March 10th, we firstly sent invitations for interview to GP trainees through a messaging platform (WeChat) introducing the background, aims and methods of the recorded, semi-structured telephone interviews. The participants who agreed to the interview were verified their eligibility and their interview dates were scheduled. Participants were interviewed using concurrent iterative analyses before saturation [9]. After 12 participants were interviewed, no new themes emerged, indicating achievement of data saturation, and hence, the recruitment was closed. A total of 25 GP trainees who were undergoing their three-year residency training program in the three teaching hospitals were invited. Three of them declined, ten were non-responders, and informed consent was obtained from 12 GP trainees who agreed to the invitation. None of the participants were familiar with the interviewers. The written consent forms of participants were scanned and emailed to the research team.

The demographics of 12 participants are presented in Table 1. The cohort, aged 22–36 years, comprised of three males. Ten participants were graduates with a Bachelor’s degree, and the other two participants held a Master’s degree. Two participants had work experience of general practice before attending the GP training program.

**Table 1:** Demographics of the participants

Paticipants code	Gender	Age (years)	Year of training	Educational background	Work experience and Departments	Workplace location
1	Male	24	1st	Bachelor of Clinical Medicine	None	Northern China
2	Female	36	1st	Graduate of General Practice	14 years as a GP	Northern China
3	Female	22	1st	Bachelor of Clinical Medicine	None	Western China
4	Female	27	3rd	Graduate of General Practice	None	Eastern China
5	Female	24	1st	Bachelor of Clinical Medicine	None	Western China
6	Male	24	1st	Bachelor of Clinical Medicine	None	Western China
7	Female	29	3rd	Bachelor of Clinical Medicine	3 years as a GP	Northern China
8	Female	24	1st	Bachelor of Clinical Medicine	None	Western China
9	Female	27	1st	Master of General Practice	1st year as a GP	Eastern China
10	Female	28	3rd	Bachelor of General Practice	None	Eastern China
11	Female	27	1st	Master of General Practice	1st year as a GP	Eastern China
12	Male	24	1st	Bachelor of Clinical Medicine	None	Western China

*GPs* General Practitioners

### Interview and data collection

The interview outline was developed specifically for this study and has not previously been published elsewhere (Additional file 1). The draft of the interview outline was developed based on literature review and opinions of experts (YZ, XZ), who had a great deal of clinical experience in general practice and participated in general practice education in GP training program and qualitative studies. The interview outline was suggested by the experts to include introduction to the study, personal information, the experiences and feelings during the epidemic, as well as the participants’ career perspectives before and after the outbreak. Open questions were designed for the participants to express their feelings and thoughts freely.

Semi-structured telephone interviews instead of face-to-face conversation were conducted between March and April 2020, considering the isolation requirement during the epidemic. All the interviews were conducted in Chinese by the experienced, trained interviewer (YY) who had previously participated in a qualitative study using the method of semi-structured interview. The interview outline was pilot tested with two participants before data collection commenced to ensure the questions were appropriate and clear. No substantial changes were made in the interview questions after pilot interviews, instead, the question order was suggested to get adjusted. Specifically, though personal factors before COVID-19 could influence their career choice, this study focused more on GP trainees’ career perspectives after the outbreak of COVID-19, as a result, we discussed the epidemic-related questions at first, and simplified their rotation experiences and career choices before the epidemic. Each interview lasted 20–53 min.

### Data analysis

Interviews were digitally recorded, transcribed verbatim in the original language (Chinese) by three of the researchers (XH, YC, HD), and the transcripts were checked for accuracy by one researcher (YY) and anonymized subsequently [10], and were then translated into English by professional translators (YC). Data were analyzed inductively by thematic analysis [11] based on the principles of grounded theory [12]. Under the supervision of the two main researchers (YZ, XZ), two researchers (YY, XC) initially read and analyzed all transcripts individually and conducted the initial data coding, which were formulated by iterative analyses, and new codes were added to the entire data set until data saturation was achieved [13]. Similar codes were grouped to form broader themes by constant comparison until themes and subthemes were developed. The themes and analyses were further discussed among research team members to come to a consensus. Finally, NVivo qualitative data analysis software (Version 10; QSR International) [14] was used for indexing and charting of the data based on the finalized coding framework.

## Results

After the outbreak of COVID-19, only one participant changed the career choice as the participant was highly interested in the specialty of Obstetrics and Gynecology. The other 11 participants were still interested to become a GP, among whom five were passionate and determined to dedicate themselves to becoming a GP after the outbreak.

Four main themes influencing GP trainees’ career perspectives after COVID-19 emerged from the interview: changes of GPs’ work content in COVID-19, challenges of being a GP, psychological changes of the career, how to provide better primary care. The categories and factors are presented in Table 2 and the participant quotations identified by participant number are described below.

**Table 2:** Categories and factors influencing participants’ perceptions of career development

Categories	Factors
**1. Changes of GPs’ work content in COVID-19**	Different responsibility of GPs in COVID-19
	New roles of GPs in COVID-19
	Increasing use of telemedicine
**2. Challenges of being a GP**	Exhaustion due to medical workloads
	Low income
	Lack of resources in primary medical institutions
	Distrust of GPs in society
**3. Psychological changes of the career**	Feeling unsafe on the job
	Inspiration by role models
	Motivation by medical colleagues
**4. How to provide better primary care**	Strengthening public health education
	Improving medical skills
	Self-protection in preventing infectious diseases
	Construction of family doctor team and better co-operation

*GPs* General Practitioners

### Changes of GPs’ work content in COVID-19

During the outbreak, GPs across the country have empowered the community by building a firewall against the deadly virus through a series of work, including case identification, disease surveillance, and health education about the epidemic, which broke the chains of transmission through early intervention. And these have been the priority of work for GPs during the epidemic. Besides, they were still providing continuing care for patients with non-communicable diseases, which was their routine work before the epidemic.

*“Some GPs worked around the clock with security staff at the checkpoints, where they recorded epidemiological history and conduct health checks for travelers. For residents in the community, health education was delivered and body temperature was recorded daily, which relied on the door-to-door visit of GPs, especially in rural areas.” (Participant 10)*

*“We also monitored the condition of patients with chronic diseases and instructed them about medication.” (Participant 2)*

During the epidemic, GPs were regarded as “gatekeeper”. Specifically, they stood sentinel to prevent the outbreak of COVID-19 in the community by performing the first checks to achieve early detection, reporting, quarantine and control. This also reduced the pressure of hospitals, for less patients flocking to hospitals.

*“GPs made contributions in identifying suspected cases, controlling the source of infection, and cutting off the route of transmission. They were seen as the health care “gatekeeper”. Their work is ordinary but crucial.” (Participant 11)*

*“The specialists are committed to reducing the number of critically ill patients and deaths, while GPs are aiming at reducing the number of infected patients and protecting the health of the whole people, which relieves the burdens of the most front-line specialists.” (Participant 4)*

Social-distancing during the epidemic presented some challenges to face-to-face communication between doctors and patients. However, the utilization of science and technology may help solve the social-distancing restrictions. Telemedicine has been getting widely used during the epidemic, which provided prevention and treatment guidance, remote medical and psychological consultation for the community residents. It is also a platform covering COVID-19 updated information for education and triaging of patients for medical staff.

*“Communication tools such as WeChat and some APPs for online medical care came into use in medical practice. Patients could make appointments and communicate with GPs online, then they were offered guidance on health care and psychological support, and purchased drugs online under the instructions of doctors, which may be a new way of work for GPs.” (Participant 9)*

*“Telemedicine provided a tracking system for infected patients and their contacts, which was convenient for disease control in community. For medical education, teaching was also shifted to digital solutions. (Participant 1)*

### Challenges of being a GP

During the epidemic, the workload of GPs was increased, which brought tremendous stress to the participants. This was mentioned by half of the participants.

*“Door-to-door visits for follow-up and health education were required every day, so the workload suddenly increased. The most prominent feeling of our colleagues was ‘very, very tired’.” (Participant 7)*

During the outbreak, though GPs’ workload got increased and they worked for longer time, their income did not increase. In fact, the income was also lower than that of other specialists, which disappointed them a lot and had negative effects on their enthusiasm in work.

*“The subsidies during the epidemic was only 1 / 12 of that for the same hours of other volunteers. It’s not a matter of money. I just don't think our efforts were appreciated.” (Participant 10)*

Some participants mentioned the lack of resources in primary medical institutions, including qualified medical staff, medications, and equipment, which limited the performance of medical workers during the epidemic.

*“The available categories of medications for chronic diseases are only a few, and computed tomography (CT) has not been popularized in primary medical units, so we can do limited things when making the diagnosis and treatment of COVID-19.” (Participant 4)*

Though a lot contributions were made by GPs during the epidemic, there was still a widespread distrust on GPs as well as doubts about their professional skills, which impeded the development of general practice in China and the enthusiasm of GPs.

*“In their opinions (including the managers), GPs can only treat minor ailments and are incompetent when it comes to complicated disease conditions. We do more managerial work than practicing medicine, thus we have little opportunities to get improved.” (Participant 10)*

### Psychological changes of the career

The outbreak event had different degrees of impact on the psychology of GP trainees as well as their families, which affected their career choice.

There was no doubt that medical staff was at high risk of encountering potentially COVID-19 infected individuals, and the sudden outbreak of the epidemic led to a shortage of medical supplies. Thus, the lack of protective equipment aggravated the situation, due to which medical staff had to face hazardous occupational exposure.

*“It is impossible to see without fear that medical staff got infected. If I can't even protect my life, what is the point of work? My parents will be more worried and they don't want me to work in the most affected areas.” (Participant 8)*

In China, violence against health professionals occurs from time to time. At the very beginning of the outbreak, an emergency physician in China named Wen Yang was fatally stabbed by a patient’s son, which triggered an outpouring of anger among the public. During the epidemic, though doctors tried their best to save lives, there were still individuals who abused or even had physical conflicts with doctors for personal reasons. The violence against health professionals made participants feel unsafe on the job.

*“Doctors are at high risks of occupational exposure in Wuhan, but some patients deliberately tore off the doctor's mask, which makes me feel disappointed.” (Participant 11)*

Despite fear and worry, most of the participants stressed the positive impact of role models, especially famous doctors in China who made great contributions to the prevention and control of the epidemic.

“*Professor Zhong Nanshan is the famous expert in respiratory diseases. He alerted the human-to-human transmission of the unknown virus for the first time; Professor Wang Chen (President of PUMC) is one of the people who proposed to establish mobile cabin hospital for hierarchical diagnosis and treatment of COVID-19. They are the role models of doctors, and I want to be like him someday.” (Participant 6)*

In addition, under the unified leadership of the government, a large number of medical staff were deployed to Wuhan for rescue. The courage and dedication of these medical colleagues greatly motivated the trainees.

*“Doctors in China responded to the call without hesitation, regardless of payment or sacrifice. They are really respectable. I am eager to go to the frontline to support if I were qualified, driven by a sense of responsibility.” (Participant 9)*

### How to provide better primary care

Several participants mentioned the poor awareness on prevention and control of infectious disease among the public during this outbreak, which was closely related to the lack of public health education. Many participants stressed that they would focus more on disease prevention and health education among the public in future primary care.

*“At the early stage of the epidemic, some people refused to follow the inspection rule and take a quarantine, or didn’t wear masks in a proper way, which reflected the lack of public health education on communicable diseases.” (Participant 1)*

*“Poor personal hygiene is not rare in rural areas. We can't wait until another epidemic to take actions. Instead, health education is necessary to keep protecting the public.” (Participant 5)*

Inspired by the medical staff in the frontline, participants generally believed that they were supposed to improve the medical skills required for disease prevention, case identification, and continuing health care. What’s more, the epidemic brought about a series of mental health problems including panic, anxiety, stress. Thus, psychological counseling will be essential and important. In the future work, more attention will be paid to psychological counseling.

*“COVID-19 is a disease with several systems and organs involved. Patients are needed to be taken care of as a whole with the help of multidiscipline cooperation, and that’s what GPs are good at. Thus, comprehensive improvement of medical skills in diagnosis and treatment of diseases are needed.” (Participant 2)*

Several participants mentioned that regular training on infectious diseases was needed in GP education. Also, they would raise more awareness on self-protection and strictly follow the regulation related to prevention of infectious disease in their daily medical work.

*“We studied knowledge of epidemics and skills for self-protection in work, such as regulations of working in the isolation area during the epidemic, and how to put on and take off the protective suit. These trainings should be a necessary part of future GP education.” (Participant 12)*

*“I am more aware of the importance of self-protection. Moreover, when we engage patients, medical history taking should be attached more importance to.” (Participant 3)*

GPs are the key roles in the battle of COVID-19 in community, however, they need to cooperate with other professionals and authorities to ensure appropriate actions have been taken to reduce risks. In addition, it was mentioned that the family doctor team with more reasonable structure were needed, and cooperation among the team members would bring about better health care to residents.

*“GPs are requested to cooperate with public health workers, community workers, volunteers, drivers, etc. to take actions in the control of infectious diseases. The cooperation is of vital importance for the anti-epidemic work being carried out smoothly in community.” (Participant 10)*

*“At present, the family doctor team is mainly composed of two to three people, including community workers, nurses and family doctors. However, if psychologists and pharmacists could be recruited, the team structure will be more reasonable, and team members will provide health care in different aspects.” (Participant 1)*

## Discussion

### Summary

The current study explored GP trainees’ career perspectives after COVID-19. Overall, no substantial effects were seen in the career choice of GP trainees, as the majority of the participants chose to continue to be a GP even after the COVID-19 outbreak. Approximately half of the participants strengthened their determination to dedicate themselves to this career, and only one participant decided to change the career choice because of interest in another specialty.

We have identified four main themes related to GP trainees’ career perspectives after COVID-19 outbreak: changes of GPs’ work content in COVID-19, challenges of being a GP, psychological changes of the career, how to provide better primary care. Although some negative effects caused psychological changes, most of participants were inspired by the role models. After the outbreak of COVID-19, GP trainees had more in-depth understanding of GPs’ work and responsibility in an epidemic outbreak, and exhibited intensions for self-improvement in career development, especially in public health education and self-protection in preventing infectious diseases. Nonetheless, there are some challenges of being a GP after COVID-19 for these trainees, which cannot be neglected.

### Comparison with existing literature

The outbreak of COVID-19 has changed the GPs’ work content and mode of work, and the participants in the current study showed an improved understanding of their work as a GP. In previous studies, there were few focusing on the effects of epidemic outbreaks on GP trainees’ career perspectives. Like a “hidden curriculum”, the influence of COVID-19 outbreak on GP trainees’ career perspectives can be positive as well as negative [15].

According to previous studies, there is much anxiety, fear and bereavement amongst GP team members at the present time not only due to increased mortality rate caused by the virus, but also related to increased risk of severe complications, changes in professional practice concerns with loss of face-to-face human connection [16]. Moreover, under the COVID-19 pandemic, physicians caring for COVID-19-infected patients have been endangered by the risk of contagion and possible mortality. Actually, the deaths of medical staff have been increasingly reported worldwide [17], and the lack of personal protective equipment was considered as a common cause [18]. These negative psychological changes were also mentioned by the participants in our study as “feeling unsafe on the job”. In view of this point, the sufficient supply of personal protective equipment and the education of how to use it [19] can greatly relieve their negative psychological changes. Also, the effective collaborative team working and understanding from friends and families can be helpful [16].

However, in this study, no substantial changes were seen in the career choice of the GP trainees before and after the epidemic, as most of them were determined to provide primary care for the public after the three-year training program. They graduated with an aspiration for a GP career [20] and hence, are less prone to negative comments owing to a mature mindset [21]. In previous studies of career choice for medical students or foundation doctors, having inspirational teachers or role-models during training was a factor in choosing general practice [22, 23]. In this epidemic, a large number of Chinese medical staff including GPs supported the epidemic areas out of responsibility, and the GP trainees in our study were also inspired by the role models and colleagues, which had a great impact on their career perspectives.

GPs played a significant role in providing health care during the COVID-19 pandemic. On one hand, they continued providing whole-person and patient-centered care to residents [24], for instance, patients with chronic diseases were encouraged to keep contact with GPs in order to avoid interruption of follow-up and breaks in long-term treatments [25]. More importantly, GPs undertook new responsibility and roles during the crisis. In France, the National Academy of Medicine recommended to place the general practitioner at the heart of the strategy for detecting new cases of COVID-19 and tracking contact subjects [25]; In UK, over 90% of COVID-19 cases were managed in primary care and the community [16]. In China, GPs in primary care institutions are engaged in guarding the checkpoints and protecting the community to curb the spread, performing detection and treatment from door to door in the community, and providing continuous care to discharged patients and the chronically ill [5]. In our study, GP trainees also took part in clinical practice during the epidemic. The work content of Chinese GPs has changed significantly, which made them realize the responsibilities and roles of GPs in an epidemic outbreak, and promoted the understanding of their career development as GPs.

During the epidemic, the restrictions of social distance significantly impacted on the face-to-face primary care consultations [16]. The increased use of technology for communication has a critical role in remote consultation and communication, and is an ideal model for managing communicable diseases [26], including telephone, video, online consultations, and some social media platform [16]. In addition, many different forms of telemedicine systems [8, 27, 28] have been deployed for GPs working in different situations such as dynamic risk assessment, triaging of patients, and following up with patients who are suspected and discharged [8],which relieved the shortage of medical resources due to its convenience to both residents and GPs [8]. As a result, the increasing use of telemedicine may change the work ways of GPs in clinical practice, as GP trainees in our study have mentioned. However, though the option of remote consultation can improve accessibility for many, it can be difficult for marginalized groups of people in society. In addition, the potential risks to patient safety and impacts on collaborative working of telemedicine also need to be paid attention to [16].

In previous studies, some factors were viewed negatively in choosing general practice as a career for medical students. The increased workload and overtime [29] make the career less appealing, and general practice was believed to be the toughest job and under increasing pressure [21]. This became more prominent during the epidemic, as GPs’ workload was higher due to increasing patients and shortage of medical staff [16, 30], which was also mentioned by the GP trainees in our study. Moreover, the low income also made students hesitate to become a GP [31–33]. The lack of respect to GPs from other doctors was considered as a “stigma” [34], and the “badmouthing” or “banter” also made medical students alter their career choices [35, 36], which were consistent with our findings. In addition, inequalities according to region and deprivation has effects on the work state of GPs. Those who worked in areas of social deprivation received minimal additional resources in spite of the extra workload [37]. In the current study, distrust of GPs in society and lack of resources in primary medical institutions were recognized as challenges to be a GP among the GP trainees. Above all, owing to the challenges faced by the GP trainees, measures are required to be needed to improve the work environment for GPs.

After the epidemic, GPs fulfilled the supportive role as public health educators through different media [24], and provided more psychosocial support to patients in alleviating their fear of being infected and keeping maintenance of their wellbeing [24]. These changes in GP’s work has influenced the career perspectives of GP trainees in our study, for they would strengthen public health education more in the future work and construct more psychological counseling with residents. Moreover, the GP trainees in our study would attach more importance to self-protection in preventing infectious diseases in their future work. A previous review also stressed the importance of appropriate use of personal protective equipment for front-line GPs [19]. Medical education about self-protection and regulations related to communicable diseases for GP trainees should be strengthened in case of future outbreaks of respiratory tract infections.

## Conclusions

The COVID-19 outbreak has brought GP trainees new career perspectives. Most of them were inspired and had an in-depth understanding about the GP’s work and responsibility during an epidemic. There were also challenges for being a GP and they had new perspectives on career development. As some medical staff were infected due to inappropriate personal protective measures, self-protection and strict observation to the regulations of infectious diseases will be stressed in the future work. As a result, training for control of communicable diseases should be considered as a necessary part of GP education in the future. Moreover, more public health education in disease prevention and constructing the family doctor team with more reasonable structure in the future are recommended.

The epidemic will definitely be under control in the near future, but the changes of GP trainees’ career perspectives during the epidemic will have an impact on their own future work. It will not only affect the quality of the primary care they provide, but also affect the GP education and development of general practice in the future. As the COVID-19 epidemic has swept across the world, we hope the experience of GP trainees in China during the epidemic may offer useful advice to other areas which are also affected.

### Strengths and limitations

Although the sample size (*n* = 12) of our study was limited, it was adequate for a qualitative study [9]. We conducted the interviews through telephone, which may encourage respondents to be more open about sensitive topics [38]. Nevertheless, this study has some limitations. The participants were GP trainees who are qualified doctors and had chosen general practice as their future career. For them, continuing to pursue a career in general practice may reflect the tendency to remain in the existing state rather than an active choice as they had invested years in the training [29] which might bias the observations that GP trainees in this study were not influenced in their career choice by the outbreak. In addition, we conducted the interviews when the outbreak was gradually brought under control, but not during the peak of the epidemic or when the epidemic has completely eased, which might create different observations.

## Supplementary Information

**Additional file 1.**

## Data Availability

The anonymised transcribed interviews from the current study are available from the corresponding author on reasonable request.

## Abbreviations

GP: General Practitioner
COVID-19: Coronavirus disease 2019
