# Supplementary material for: General practitioner trainees’ career perspectives after COVID-19: a qualitative study in China
Source: BMC Fam Pract. 2021 Jan 11;22:18. doi: 10.1186/s12875-020-01364-x (PMC7797889; doi:10.1186/s12875-020-01364-x)
Supplement: Supplementary file 1 — Additional file 1. [file 12875_2020_1364_MOESM1_ESM.docx]

**Additional file: Interview guides**

A translated edition of the interview outline centered on the participants’ experiences and feelings during the COVID-19 epidemic, as well as their perspectives about the career, which was developed specifically for use in this study and has not been published before.

| **Introduction** | ▪ Information about the interviewer.  ▪ Introduction of the study and the aims: The COVID-19 outbreak  has become a major public health event since the beginning of the  year 2020, and General Practitioners (GPs) made contributions in  disease control. This study is to explore GP trainees’ career  perspectives after COVID-19.  ▪ Tell the participants about the confidentiality and anonymity.  ▪ Information about the structure of the interview. |
| --- | --- |
| **Demographics of the participants** | 1. Gender, age, year of training, educational background, work  experience and departments, workplace location. |
| **Experiences during COVID-19** | 2. As a GP trainee, did you take part in the work fighting against the  virus? |
|  | ▪ Follow up: Please introduce your daily work briefly. |
|  | 3. What do you know about the roles of GPs in COVID-19 through  your work experience or reported news on social media? |
|  | ▪ Follow up: Can you give some examples? |
|  | 4. Which events have made a deep impression on you during the  outbreak? |
|  | ▪ Follow up: 🢭 As a GP trainee, how do you feel about these  events? |
|  | 🢭 What are the positive and negative effects of  these events on your career perspective? |
| **Career choice before COVID-19** | 5. Now let’s look back at the time before the outbreak. Why did you  choose to participate in the GP training program? |
|  | ▪ Follow up: Were you willing to be a GP back then? |
| **Career choice and perspectives after experiencing the outbreak** | 6. After the outbreak, did you make any changes in your career  planning/career choice in the future? |
|  | ▪ Follow up: What has caused that change? |
|  | 7. How much did other people’s ideas and comments affect the way  you think or used to think about your career planning? |
|  | ▪ Follow up: Can you give me some examples? |
|  | 8. After the COVID-19 outbreak, what do you expect about the  future of the profession? |
| **Closing** | 9. Are there something you want to add, something important we  have not talked about concerning your perspectives about the  career? |
